# Beyond the Cortico-Centric Models of Cognition: The Role of Subcortical Functioning in Neurodevelopmental Disorders

**DOI:** 10.3389/fpsyg.2019.02809

**Published:** 2019-12-11

**Authors:** Flavia Lecciso, Barbara Colombo

**Affiliations:** ^1^Lab of Applied Psychology and Intervention, Department of History, Society and Human Studies, Università del Salento, Lecce, Italy; ^2^Neuroscience Lab, Champlain College, Burlington, VT, United States

**Keywords:** cortical, subcortical, cognition, ADHD (attention deficit and hyperactivity disorder), ASD, Rett, SLI

## Introduction

The main aim of the present opinion article is to discuss and argue how the classic cortico-centric model of neurodevelopmental disorders is not exhaustive of the possible explanation of these disorders. The alternative proposal presented here is to include the cortico-subcortical network model to explain them.

### The Role of a Distributed Cortical Network in Neurodevelopment

Literature focusing on typical and atypical neurodevelopment has been reporting more and more frequently how human cognition relies on a distributed cortical network (e.g., Jasinska et al., 2012). This network includes the dorsolateral prefrontal cortex, dorsomedial prefrontal cortex, anterior cingulate cortex, inferior frontal gyrus, the insula, and parietal regions (Ishai, 2008; Petersen and Sporns, 2015). The cortico-centric model has been the most used approach to explain the human cognitive functioning from pathology to normal development (Antonietti et al., 2005; Fabio et al., 2005, 2018a, 2019e; Koziol and Budding, 2009; Fabio and Towey, 2018; Caprì et al., 2019). However, traditional cortico-centric tend not to show a coherent and unite neuroanatomic framework that considers the overlapping levels of cognitive-control functions. This is caused by the fact that they highlight the contributions of the prefrontal cortex in neurodevelopment, while they do not consider the role of subcortical brain structures. In young children, the cortex is immature and, consequently, subcortical processes are dominant (Koziol and Budding, 2009).

### The Role of Cortical and Subcortical Networks

Recently, it has been proved that the cortex, basal ganglia, and cerebellum play a crucial role both in the typical development of functional brain and neurodevelopmental disorders (Ouhaz et al., 2018). Thus, we argue that different neurodevelopmental disorders as attention deficit hyperactivity disorder (ADHD), autistic spectrum disorders (ASD) as well as several other learning disabilities can involve primarily cortical and subcortical networks. For example, MRI data have shown structural alterations both in cortical and subcortical brain regions in children and adults with ADHD (De La Fuente et al., 2013; Fabio and Caprì, 2017; Fabio et al., 2018c). Consistent abnormalities were found in the fronto-striatal, fronto-temporoparietal, and fronto-cerebellar networks, which were developing late. In addition, several frontal brain regions (including parieto-temporal areas, the basal ganglia, posterior cingulate, the cerebellum and the splenium of the corpus callosum) have been found to have reduced volume and cortical thickness (Hoogman et al., 2018). These cortical-subcortical networks are known to mediate the automatic and controlled processing in human cognition (Fabio and Caprì, 2015, 2019; Fabio, 2017).

Despite this evidence ADHD is considered to be cortically based. This means explaining it in terms of frontal cortex dysfunctions and executive deficits.Only a few studies have examined the role of subcortical brain structure and automatic processes in ADHD. Some data reported in literature highlight how children with ADHD tend not to perform as well as typically developing children in tasks that requires the activation of automatic and/or more controlled processing strategies (Fabio and Urso, 2014; Fabio et al., 2019b). Other studies reported abnormalities within specific cerebellar-prefrontal circuitry. These abnormalities are supposed to help explaining some of the typical ADHD symptoms (Seidman et al., 2005). Moreover, ADHD children have been recently found to have a deficit in the automaticity process (Fabio et al., 2015; Martino et al., 2017).

At the same time, literature reports data to prove that children with specific language impairment (SLI) show brain abnormalities linked to procedural memory, specifically in the frontal/basal-ganglia circuits and the cerebellum (Koziol and Budding, 2009). These brain dysfunctions can explain their language problems. In general, when attention problems prevent or avoid the proceduralization of basic cognitive processes, it is likely that the brain structures underlying procedural memory are affected. Besides, as compared to the typical developmental children, the children with SLI and children with Rett Syndrome (RS) present procedural memory impairment (automatic deficits), even when holding working memory constant (Fabio et al., 2018b,e, 2019a,d; Gangemi et al., 2018). Thus, SLI and ADHD and RS can be conceptualized as characterized by impairments within cortico-striatal procedural learning systems (Fabio et al., 2011, 2016; Castelli et al., 2013).

Following this line of reasoning, we can argue that ASD can rely on a distributed cortical and subcortical network. Neuroimaging studies reported metabolic deficits in individuals with ASD, compared to typical developmental subjects. These deficits affect several areas including cortical areas like frontal and temporal cortex, and subcortical areas like basal ganglia and the cerebellum (Rumsey and Ernst, 2000). To be more specific, extensive MRI studies exploring volumetric differences and intercorrelations in subjects with ASD compared with healthy subjects matched by ages, showed distinct differences between groups. Data highlighted abnormalities for the clinical sample in both the anatomy and connectivity of limbic–striatal systems (McAlonan et al., 2004). These parts of the brain are considered the basis of social behavior, so the differences highlighted through these studies could explain both brain metabolic and behavioral differences in ASD. A recent study (Conti et al., 2017) followed up on this line of research by comparing toddler with ASD to toddlers with other developmental disabilies. Data highlighted that children with ASD showed clear patterns of overconnectivity both in cortical (fronto-temporal nodes, related to social-skill development) and subcortical (basal ganglia, related to repetitive behaviors) networks.

Abnormalities of cortical and subcortical structures have been observed also in genetic syndromes (Fabio et al., 2018d,f, 2019c; Martino et al., 2018). For example, it was found that individuals with Tourette's syndrome show differences in blood oxygenation levels in several regions including prefrontal, temporal, and parietal cortices and also basal ganglia and thalamus (Polyanska et al., 2017; Jo et al., 2018), as well as an increased activation of the primary and supplementary motor fronto-striatal circuits. It is known that an activation of the temporal and parietal cortical is required for tic suppression (Loo et al., 2019) and that the activation of motor and language circuits can explain vocal and motor tics (Stern et al., 2000).

## Discussion

In literature, several studies have suggested that the basal ganglia and the cerebellum play an essential role in neurodevelopment and especially in cognitive and emotional functioning (Harris, 2003; Arnsten and Rubia, 2012). Consequently, when we try to get a deeper understanding of human cognitive or non-cognitive functioning, we should take into consideration the influence played in the opposite direction by cortical and subcortical structures. This means to consider that the subcortical structures, traditionally related to motor control, are involved in functions that are not related to motor functions (motor control or planning). Thus, we want to highlight that behaviors can be distributed at cortical and subcortical levels. It is crucial to highlight that the posterior cerebral cortices are primarily involved in sensorial processing. At the same time, the prefrontal and frontal cortices are involved in mechanisms that deal with programming behaviors, while the basal ganglia are relevant to attention processes and action selections. These selections are used by the cerebellum to determine if and how an behavior should be amplified. This choice depends on the sensory-perceptual context and specific goals or purposes of each individual. Starting from distinct goals, the cerebellum establishes specific control models that allow for optimal manipulation of motor and cognitive activities. To enable this process to be smooth, these three brain regions have to work synergetically and in coordinate if unique ways. Having a good understanding of this relationship between cortical and subcortical structures has an important implication because it fosters a change of perspective from a cortico-centric model of brain-behavior relationships to a cortical and subcortical model of neurodevelopment disorders.

## Author Contributions

FL and BC researched existing literature, discussed the topic, and wrote the paper.

### Conflict of Interest

The authors declare that the research was conducted in the absence of any commercial or financial relationships that could be construed as a potential conflict of interest.
